# Does *S*-Metolachlor Affect the Performance of *Pseudomonas* sp. Strain ADP as Bioaugmentation Bacterium for Atrazine-Contaminated Soils?

**DOI:** 10.1371/journal.pone.0037140

**Published:** 2012-05-15

**Authors:** Cristina A. Viegas, Catarina Costa, Sandra André, Paula Viana, Rui Ribeiro, Matilde Moreira-Santos

**Affiliations:** 1 Department of Bioengineering, Instituto Superior Técnico (IST), Technical University of Lisbon (TUL), Lisboa, Portugal; 2 Institute for Biotechnology and Bioengineering (IBB), Centre for Biological and Chemical Engineering, Instituto Superior Técnico (IST), Technical University of Lisbon (TUL), Lisboa, Portugal; 3 Instituto do Mar (IMAR), Department of Life Sciences, University of Coimbra, Coimbra, Portugal; 4 Agência Portuguesa do Ambiente (APA), Amadora, Portugal; University of Kansas, United States of America

## Abstract

Atrazine (ATZ) and *S*-metolachlor (*S*-MET) are two herbicides widely used, often as mixtures. The present work examined whether the presence of *S*-MET affects the ATZ-biodegradation activity of the bioaugmentation bacterium *Pseudomonas* sp. strain ADP in a crop soil. *S*-MET concentrations were selected for their relevance in worst-case scenarios of soil contamination by a commercial formulation containing both herbicides. At concentrations representative of application of high doses of the formulation (up to 50 µg g^−1^ of soil, corresponding to a dose approximately 50× higher than the recommended field dose (RD)), the presence of pure *S*-MET significantly affected neither bacteria survival (∼10^7^ initial viable cells g^−1^ of soil) nor its ATZ-mineralization activity. Consistently, biodegradation experiments, in larger soil microcosms spiked with 20× or 50×RD of the double formulation and inoculated with the bacterium, revealed ATZ to be rapidly (in up to 5 days) and extensively (>96%) removed from the soil. During the 5 days, concentration of *S*-MET decreased moderately to about 60% of the initial, both in inoculated and non-inoculated microcosms. Concomitantly, an accumulation of the two metabolites *S*-MET ethanesulfonic acid and *S*-MET oxanilic acid was found. Despite the dissipation of almost all the ATZ from the treated soils, the respective eluates were still highly toxic to an aquatic microalgae species, being as toxic as those from the untreated soil. We suggest that this high toxicity may be due to the *S*-MET and/or its metabolites remaining in the soil.

## Introduction

Diverse pesticides (and their metabolites), fertilizers and organic components used in commercial formulations can be found in agricultural soils due to intensive use in crop cultivation or accidental spills. In particular, agricultural dealership sites and mix-load or disposal sites may represent potential sources of environmental contamination with high levels of diverse compounds [1], [2]. For example, 205, 2272, 13, 1829 and 108 µg g^−1^ of soil of the *s*-triazine herbicides atrazine (ATZ), cyanazine, simazine and the chloroacetanilide herbicides metolachlor (MET) and alachlor, respectively, were measured in soil samples from a mix-load site [1]. As a consequence, mixtures of chemicals are likely to contaminate freshwaters via leaching, drainage and runoff events [3]–[5], and toxicological synergies of multiple chemicals may enhance deleterious effects on ecosystems and human health [6]. Herbicidal formulations containing ATZ, either as the sole active ingredient or in combination with the racemic mixture of MET or, more recently, with the product enriched in the active *S*-enantiomer (*S*-MET), have been used worldwide for broad-spectrum weed control in several crops [7]–[10].

In soil, ATZ presents moderate persistency (DT_50_ = 28–150 days, field, aerobic) and mobility (soil organic carbon/water partition coefficient, K_OC_ = 100 L kg^−1^) [8]. These properties and increasing concerns regarding potential impact of ATZ and its toxic chlorinated N-dehalkylated metabolites on human health and ecosystems [11]–[13], promoted active research on ATZ-degrading microorganisms and on bioremediation strategies aiming to reduce soil contamination to safe levels and to minimize dispersion into surrounding aquatic compartments [1], [14]–[19]. *Pseudomonas* sp. ADP is the best-characterized ATZ-mineralizing bacteria and uses ATZ as sole N source by means of a catabolic pathway encoded in the plasmid pADP-1 [16], [18]. It has high potential for the bioaugmentation of ATZ-contaminated soils, but, presumably due to C limitation and low survival in soil, it was found to be less effective at high ATZ concentrations that are relevant in the case of spill or careless disposal scenarios [17], [18]. As part of a framework for the rational bioremediation of ATZ-contaminated land, we recently presented evidences that a clean-up strategy, combining soil bioaugmentation with this bacterial strain and biostimulation with citrate [17], was effective at a larger microcosms scale [14], [15]. It led to the rapid removal of ATZ from a natural crop soil spiked with a commercial formulation containing ATZ as single active ingredient at doses mimicking worst-case scenarios (e.g. spills and/or concentrated hotspots) [14], [15]. More importantly, assessment of the ecotoxicity of soil samples and eluates/leachates from the bioremediated soils proved the effective soil decontamination in less than 10 days, hence contributing to significantly diminish the toxicity impact in the aquatic compartment [14].

**Figure 1:**
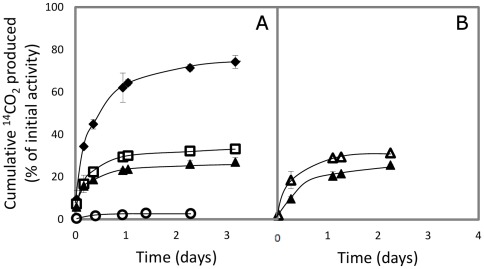
ATZ mineralization by *Pseudomonas* sp. ADP Rif^R^ in soil contaminated with Primextra S-Gold. Time-course formation of ^14^CO_2_ from [ring-UL-^14^C]ATZ in (**A**) soil spiked with [^14^C]ATZ plus increasing doses of Primextra S-Gold as follows: 5× (⧫), 20× (□) and 50×RD (▴), without citrate amendment; or (**B**) soil spiked with [^14^C]ATZ plus 50×RD of Primextra S-Gold and amended with 3.4 mg g^−1^ trisodium citrate (Δ) or non-amended (▴). The average amount of ^14^CO_2_ released from non-inoculated control soil is shown for comparison (○) in (**A**). Data are means±SD of measurements from three replicated samples from at least two independent experiments under each condition.

*S*-metolachlor is relatively non-persistent (DT_50_ = 21 days, field, aerobic) and moderately mobile (K_OC_ = 226 L kg^−1^) in soil, but very persistent in water [8], [9]. Both the racemic mixture and the *S*-enantiomer have the potential to cause adverse effects on microorganisms [2], [20] and on higher terrestrial and aquatic organisms [8], [20]–[22]. Freshwater macrophytes and microalgae are however the most vulnerable ones [6], [8], [23]. To our knowledge, the few published studies addressing the effect of MET or *S*-MET on ATZ-degradation in soil have been limited to indigenous soil microorganisms and intrinsic biodegradation. In some cases a negative effect was demonstrated [2] while in others there was apparently no impact [24].

Possible deleterious effects exerted by *S*-MET [2] could contribute to diminish the efficacy of the bioremediation tool previously shown to be effective for the clean-up of soils contaminated with high concentrations of ATZ [14], [15], [17]. Therefore, the present work aimed examining whether *S*-MET could negatively affect the survival and the ATZ-biodegradation performance of the bioaugmentation bacterium *Pseudomonas* sp. strain ADP in a crop soil. We used a representative crop soil from Central Portugal [14], [15] spiked with mixtures of ATZ and *S*-MET (either as the commercial formulation Primextra S-Gold or as pure active ingredients). Doses mimicked worst-case scenarios of soil contamination, being thus higher than the recommended dose (RD) for weed control in corn plantations. The following issues were addressed: first, the ability of *Pseudomonas* sp. ADP, either combined or not with citrate amendment, to mineralize [ring-UL-^14^C]ATZ mixed with increasing doses of Primextra S-Gold (up to 50×RD), in soil at small laboratory scale. Second, the effects of *S*-MET in a pure form on the bacteria survival and on its ATZ-mineralizing ability, at concentrations of the pure active ingredients representing up to 50×RD, also in small soil microcosms. Third, the rate and extent of biodegradation of ATZ from 20× or 50×RD Primextra S-Gold when applying the bioremediation tool, in larger and more realistic soil microcosms [14], [15]. The efficacy of ATZ-biodegradation in soil microcosms was evaluated mainly by performing microalgae ecotoxicity tests (72-hours *Pseudokirchneriella subcapitata* growth tests) on the eluates from soil samples. At this stage, eluates from soils contaminated with the herbicidal formulation Atrazerba FL (with ATZ as single active ingredient) and previously reported to be decontaminated after a 10-days treatment with the bioremediation tool [14] were also used for comparison purposes. The extent of ATZ removal was also examined with chemical analysis of ATZ and its metabolites in soil samples. In addition, possible modifications in the concentration of *S*-MET initially present in the soil microcosms contaminated with Primextra S-Gold as well as the possible formation of its major degradation products *S*-MET oxanilic acid (OA) and *S*-MET ethanesulfonic acid (ESA) [8], [25], [26] were also examined during the biodegradation experiments carried out.

## Results

### ATZ mineralization by *Pseudomonas* sp. ADP in soil spiked with Primextra S-Gold

Following inoculation with viable cells of the bioaugmentation bacterium (1.3±0.5×10^7^ CFU g^−1^ soil dry weight) of soil spiked with mixtures of [^14^C]ATZ plus increasing doses of non-labeled ATZ from the double formulation Primextra S-Gold (5×, 20× or 50×RD), ATZ mineralization started rapidly. The percentage of ^14^CO_2_ produced from the ^14^C-labeled ATZ attained maximal values within 3 days (Fig. 1A). Comparatively, ATZ mineralization was negligible (<2%) in the non-inoculated control soil during the same time-period (Fig. 1A). However, a moderate inhibition of rate and extent of ATZ mineralization occurred in the soils contaminated with doses of Primextra S-Gold increasing from 5× up to 50×RD (Fig. 1A). For example, the percentage of initial labeled ATZ evolving as ^14^CO_2_ at day 3 were 74.3±3.0% and 27.0±2.1% in the soil microcosms with 5× and 50×RD of Primextra S-Gold, respectively (Fig. 1A). These values correspond to approximately 37 and 24%, respectively, of the total estimated amount of ATZ mixed into the soil (assuming that labeled and non-labeled ATZ may be mineralized homogeneously). The soil that had been contaminated with 50×RD of Primextra S-Gold showed the lowest percentage of [^14^C]ATZ mineralization following bioaugmentation (Fig. 1A). In this soil, enhancement of the ratio of soluble carbon to nitrogen from atrazine (C_s_∶N_atz_) from ∼1 (in the crop soil used) up to ∼50 [15], due to soil amendment with trisodium citrate, led to a slight but significant increase in the rate and extent of ^14^CO_2_ formation (Fig. 1B).

### Effects of *S*-MET in *Pseudomonas* sp. ADP survival and ability to mineralize ATZ in soil

**Figure 2:**
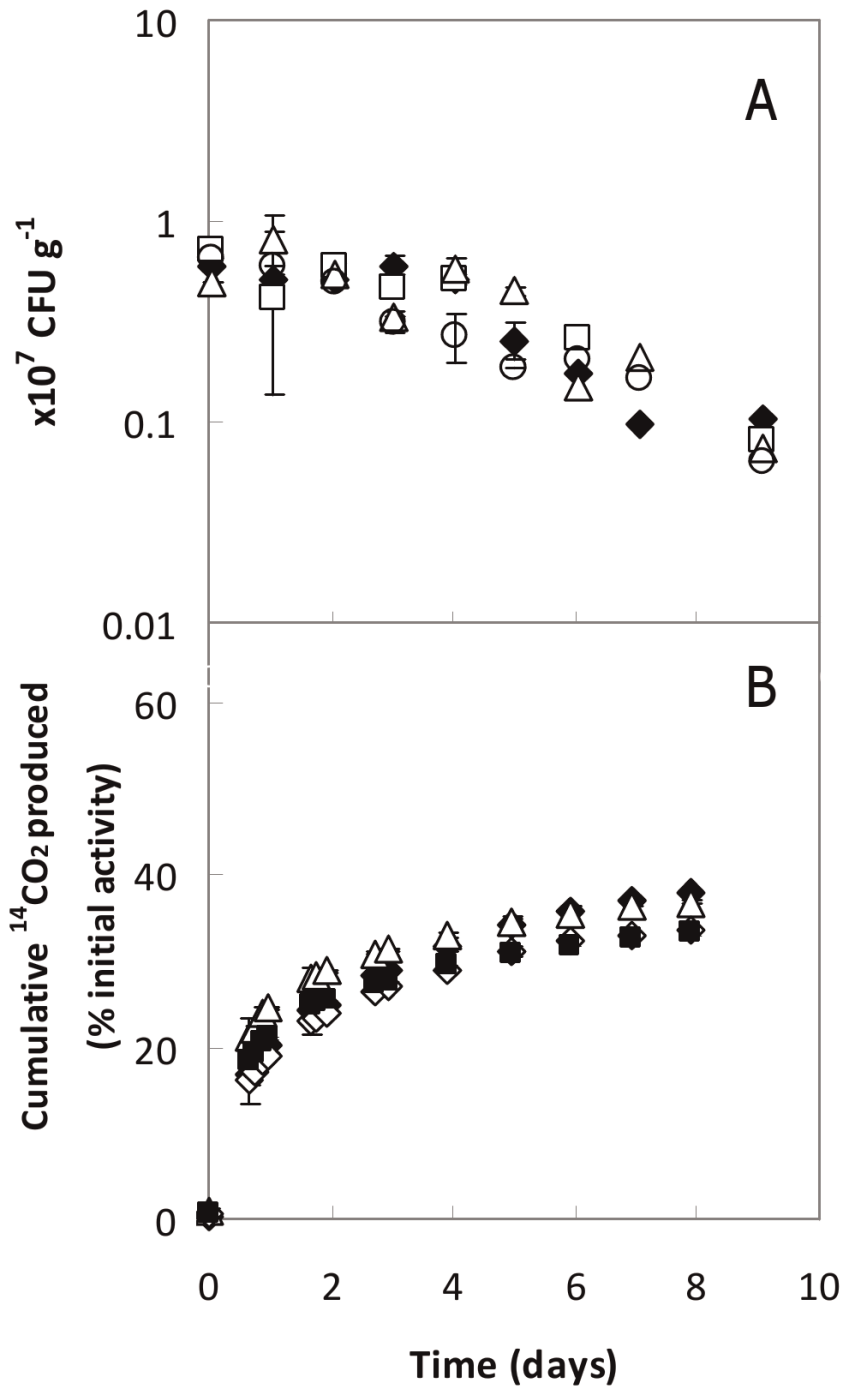
Effects of *S*-MET in *Pseudomonas* sp. ADP Rif^R^ (A) survival and (B) ATZ- mineralization in soil. In (**A**), soil previously contaminated with 24 µg ATZ g^−1^ of soil was supplemented with increasing concentrations of *S*-MET, namely 0 (⧫), 15 (□), 30 (Δ) or 60 (○) µg g^−1^ of soil, prior to inoculation. In (**B**), soil was spiked with a total of 24 µg ATZ g^−1^ of soil (including [^14^C]ATZ) plus 30 µg *S*-MET g^−1^ (Δ) or no *S*-MET (⧫), or with a total of 40 µg ATZ g^−1^ plus 50 µg *S*-MET g^−1^ (▪) or no *S*-MET (⋄), prior to inoculation. Data are means±SD of measurements from at least duplicate determinations from two or three independent experiments under identical conditions.

Viable populations of *Pseudomonas* sp. ADP inoculated into the soil were exposed to mixtures of ATZ plus *S*-MET at concentrations representing applications of approximately 30× or 50×RD of each active ingredient (Fig. 2). For the soil contaminated with 24 µg ATZ g^−1^, the addition of *S*-MET at 30 µg g^−1^ (representing ∼30×RD of each active substance) and up to 60 µg g^−1^, did not significantly affect the bacterial survival (Fig. 2A). Moreover, the rate and extent of [^14^C]ATZ mineralization was essentially the same whether or not *S*-MET was added to soil previously contaminated with a total of 24 or 40 µg ATZ g^−1^ of soil (Fig. 2B), indicating that presence of *S*-MET does not significantly affect the ability of *Pseudomonas* sp. ADP to mineralize ATZ in the worst-case conditions tested herein.

### Biodegradation of ATZ from Primextra S-Gold in larger soil microcosms

**Figure 3:**
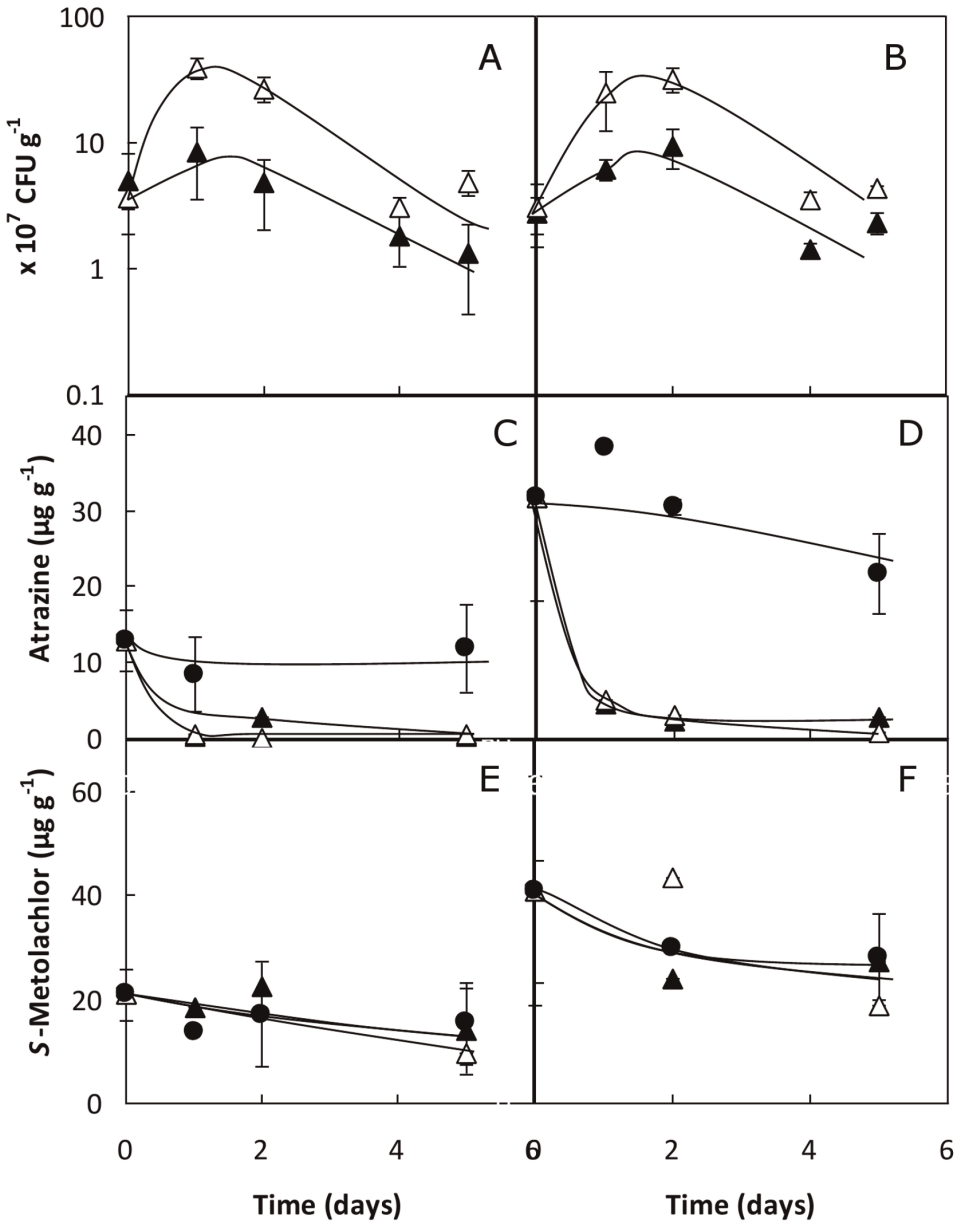
Biodegradation of ATZ from Primextra *S*-Gold and fate of *S*-MET in larger soil microcosms. Time-course variation of (**A, B**) the concentration of viable cells of *Pseudomonas* sp. ADP Rif^R^, (**C, D**) the average concentration of ATZ and (**E, F**) the average concentration of *S*-MET, in the soil microcosms contaminated with (**A, C, E**) 20×RD or (**B, D, F**) 50×RD of Primextra S-Gold, and bioaugmented with *Pseudomonas* sp. ADP with (Δ) or without (▴) citrate amendment, during incubation at 25°C. ATZ and *S*-MET concentrations in the non-inoculated control soil (•) are also shown in (C, D) and (E, F), respectively, for comparison. Data are means±SD of measurements from at least two replicated samples from two independent experiments under each condition.

The performance of the bioaugmentation/biostimulation treatment for ATZ-contaminated soils, consisting on one initial inoculation with *Pseudomonas* sp. ADP (4.1±1.2×10^7^ CFU g^−1^ soil dry weight) combined with soil amendment with trisodium citrate (C_s_∶N_atz_∼50) [15], was examined in larger soil microcosms spiked with 20× or 50×RD of Primextra S-Gold (Fig. 3). For both doses of the commercial formulation and upon soil bioaugmentation, bacterial numbers were always higher (2.5-fold, in average) in the soil amended with citrate compared with non-amended one (Figs. 3A and B). Despite that, whether or not soil was amended with citrate, most of the initial ATZ was rapidly removed from soil in up to 2 days with no lag period required (Figs. 3C and D). This high rate of ATZ biodegradation contrasted with the high levels of ATZ remaining in the untreated soils (Figs. 3C and D). Nevertheless, slight differences on the extent of ATZ biodegradation in the inoculated soils were observed depending on the initial level of soil contamination. For example, in the soil spiked with 20×RD of Primextra S-Gold, the ATZ concentration declined by ∼96%, from 12.8±0.4 to less than 0.5 µg g^−1^, in only 5 days (Fig. 3C). On the other hand, in the soil with the highest dose of Primextra S-Gold (50×RD, corresponding to an initial measured ATZ of 29.1±12.5 µg g^−1^ soil dry weight), inoculation with the bacterium without citrate amendment led to quite high levels of ATZ still remaining in the soil, namely 2.4 and 1.4±0.8 µg ATZ g^−1^ at days 5 and 8, respectively (Fig. 3D and data not shown). Combination of soil bioaugmentation with citrate amendment apparently allowed a slight but significant improvement in ATZ removal from the soil, with its concentration decreasing to 0.4±0.1 µg g^−1^ in 8 days (Fig. 3D and data not shown).

### Fate of *S*-MET and its major degradation products in the soil microcosms contaminated with Primextra S-Gold

Dissipation of *S*-MET in the soil microscosms was not different in untreated or inoculated soil (Figs. 3E and 3F). Indeed, in all the different conditions tested, its concentration was moderately reduced by around 40% on average during the first 5 days. For example, *S*-MET concentration declined from the initial 21±5 and 35±19 µg g^−1^ in the 20×RD and the 50×RD-contaminated soils, respectively, to 13±2 and 21±4 µg g^−1^at day 5 (Figs. 3E and 3F), and maintained identical values after 8 days (data not shown). This decrease in the concentration of *S*-MET was accompanied by the accumulation of its derivatives *S*-MET ESA and *S*-MET OA in the soil microcosms. Namely, *S*-MET ESA concentration increased from undetectable values (<6 µg kg^−1^ soil dry weight), at time zero, to 27±9 and 46±19 µg kg^−1^ after 5 days of incubation, in the 20×RD and the 50×RD-contaminated soils, respectively. The *S*-MET OA concentration increased from <6 to 42±22 and 87±33 µg kg^−1^, respectively, in the same period of time. At day 8, the concentrations of these compounds measured in the soil microcosms were somewhat lower, namely 22±2 and 28±7 µg *S*-MET ESA kg^−1^, or 29±5 and 43±35 µg *S*-MET OA kg^−1^, in the 20×RD and the 50×RD-contaminated soils, respectively (data not shown).

### Ecotoxicity removal efficacy

**Figure 4:**
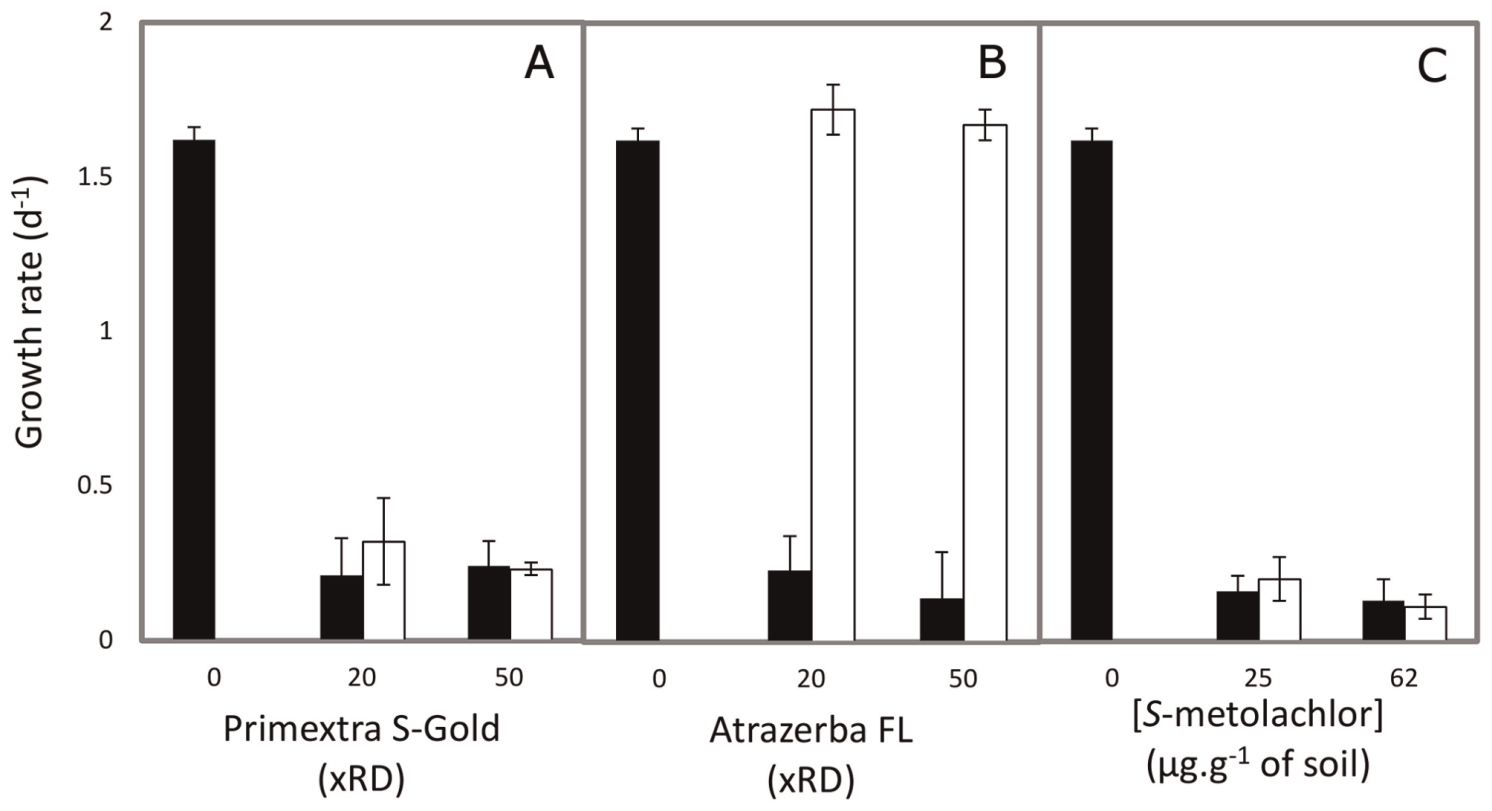
Ecotoxicity of eluates prepared from herbicide-contaminated soil. Mean 72-hours growth rate of *Pseudokirchneriella subcapitata* on eluates prepared with soil samples collected from microcosms spiked with the indicated doses of (**A**) Primextra S-Gold, (**B**) Atrazerba FL, or (**C**) pure *S*-MET, at day 5 after soil amendment with *Pseudomonas* sp. ADP Rif^R^ plus citrate (white columns) or without the bioaugmentation/biostimulation tool (black columns). Data are means±SD from toxicity tests in at least three independent eluate samples.

To estimate potential ecotoxicological effects of addition of the two doses of Primextra S-Gold (20× and 50×RD) to soil, and to assess the efficacy of treatment of these soils with the bioremediation tool, the ecotoxicity to microalgae was assessed in eluates prepared from soil samples collected in the microcosms during the biodegradation experiments with *Pseudomonas* sp. ADP plus citrate (Fig. 3), as examined before for the case of soils contaminated with up to 20×RD of the single ATZ formulation Atrazerba FL [14]. Since there was a similarity between toxicity data obtained with the eluates from soil samples collected after 5 or 8 days upon treatment with the bioremediation tool, only the 5 days toxicity data are presented in Fig. 4.

The eluates from the soil microcosms that were spiked with Primextra S-Gold but not subjected to biaugmentation/biostimulation treatment caused serious deleterious effects on microalgae growth, compared with those from soil not contaminated with the commercial formulation (Fig. 4A). After treatment with the bioremediation tool, the toxicity of the soil eluates was still significantly higher than that of the eluates from the control soil not-contaminated with the herbicide (Fig. 4A). Indeed, microalgae growth rates were similar in the eluates from the treated or untreated soils, for both doses of Primextra S-Gold (Fig. 4A). On the contrary, treatment of Atrazerba-FL-contaminated soils (up to 50×RD) with the same bioaugmentation/biostimulation tool, led to an effective decrease on the ecotoxicity of the respective eluates, compared to the eluates from untreated soil (Fig. 4B). More importantly and corroborating previous observations [14], toxicity values were achieved which were comparable to those obtained for eluates from the soil not contaminated with the herbicide (Fig. 4B).

In addition, we examined whether the *S*-MET (and/or its degradation products) that remained in the soil during the biodegradation experiments in the microcosms contaminated with Primextra *S*-Gold (Figs. 3E and F, and data described in previous section) could be responsible, at least partially, for the high toxicity of the respective eluates (Fig. 4A). Consistently, eluates from soils that were contaminated with *S*-MET in the pure form (25 or 62 µg g^−1^ soil dry weight) were also highly toxic to the microalgae irrespective of the soil having been subjected or not to the *Pseudomonas* sp. ADP plus citrate treatment (Fig. 4C).

## Discussion

### Effects of *S*-MET on *Pseudomonas* sp. ADP performance

When considering *in situ* bioremediation strategies, bioaugmentation may fail in the field as a result of the susceptibility of the specialized degrading microorganisms to high concentrations of non-target compounds; other plausible reasons may include microbial competition for a limiting nutrient and/or inhibition of degradation through catabolic repression/competitive inhibition phenomena in the soil [1], [2], [26], [27]. The significance of pesticide interactions with intrinsic xenobiotic degraders or with bioaugmentation bacteria in soil is thus a relevant issue [2], [24], [26], [27]. In the present work, we examined whether the presence of levels of *S*-MET representing worst-case scenarios of soil contamination with a commercial formulation containing both *S*-MET and ATZ may affect the performance of the bioaugmentation bacterium *Pseudomonas* sp. ADP for ATZ-biodegradation in soil. Soil amendment with viable cells of this bacterial strain plus an adequate provision of citrate to get a ratio C_s_∶N_atz_∼50, has previously been shown to be effective in the cleanup of a crop soil contaminated with high doses (up to 20×RD, mimicking similar worst-case situations) of the formulation Atrazerba FL [14]. It is worth mentioning that intrinsic ATZ-mineralization is absent or shows a very long lag-phase in the soil used ([14], [15], and this work). In the present study, we demonstrated that the presence of *S*-MET in the soil at concentrations between 15 and 50 µg g^−1^ of soil, representative of applications of up to approximately 50×RD of the double herbicidal formulation Primextra S-Gold, affected neither the survival of *Pseudomonas* sp. ADP used to bioaugment the contaminated soil (∼10^7^ CFU g^−1^ of soil) nor the rate and extent of mineralization of ^14^C-labeled ATZ by this bacterium. Consistently, its growth curve in liquid PADP medium (initial inoculum around 4×10^7^ CFU ml^−1^) was not affected as a result of medium supplementation with *S*-MET at concentrations up to 60 µg ml^−1^ (Costa C and Viegas CA, unpublished results). Based on the results obtained herein, we speculate that the observed moderate decrease in the bacterium ability to mineralize [^14^C]ATZ in the presence of increasing doses of Primextra S-Gold (from 5× up to 50×RD) may mainly reflect inhibitory effects on the bacterium physiology presumably caused by high concentrations of ATZ in soil (as suggested in Silva et al. [17]) and/or by unknown substances that may be present in the commercial formulation (even though not addressed in more detail in the present work), rather than by the active ingredient *S*-MET. Other authors [24] also reported that the pattern of intrinsic degradation of ATZ (initial concentration 40 µg g^−1^ of soil) in a soil from a pesticide-contaminated site was not significantly affected by the presence of 50 µg g^−1^ of MET or of another herbicide, pendimethalin [24]. On the contrary, Moorman et al. [2] reported that the viability of indigenous ATZ-degrading microorganisms and hence ATZ-mineralization in a contaminated soil from an agricultural chemical dealership area were negatively affected by high concentrations of MET. In the latter study, however, the authors tested the effects of MET at 200 µg g^−1^ of soil [2], which is a concentration quite higher and thus presumably more toxic than the ones tested in the present work.

Consistently, based solely on soil chemical analysis, we present evidences that in soil microcosms contaminated with 20× or 50×RD Primextra S-Gold and subsequently treated with optimized quantities of *Pseudomonas* sp. ADP and citrate, ATZ removal from soil, due to its biodegradation, was extensive (>96% of the initial, to less than 0.5 µg g^−1^ of soil) and rapid (in less than 1 week) as reported before for the soils contaminated with the formulation Atrazerba FL that contains ATZ as the sole active herbicide [14], [15]. Moreover, these results together with indications from the chemical data that, in the time-frame of the biodegradation experiments, deethylatrazine (DEA) and deisopropylatrazine (DIA) did not accumulate in the soils (data not shown), point to a significant potential reduction in the dispersion of the herbicide and of its highly toxic N-dealkylated metabolites into the adjacent water compartments, as reported before [14]. In agreement with previous studies, this would indicate an important environmental impact of the treatment of the contaminated soils with the bioremediation tool [14], [15], [18]. However, contrarily to what is reported for the case of soils contaminated with Atrazerba FL ([14], and present work), the eluates prepared from the soils spiked with Primextra S-Gold and subsequently treated with the ATZ-degrading bacterium plus citrate remained significantly toxic to the microalgae *P. subcapitata* in the time-frame of the biodegradation experiments. Based on experimental evidences, we propose that the high ecotoxicity of these eluates may be mainly associated with the presence of *S*-MET that remained in the soil and/or of its degradates, as is further discussed below.

### Contribution of *S*-MET for water extracts ecotoxicity

On one hand, soil bioaugmentation with *Pseudomonas* sp. ADP apparently had no significant effect on the dissipation of *S*-MET in the soil microcosms contaminated with Primextra S-Gold, suggesting that this bacterial strain is not able to degrade the chloroacetanilide herbicide, as reported by others [19]. In spite of that, the concentration of *S*-MET in the soils decreased moderately during the 8 days of the biodegradation experiments even though keeping values always higher than 60% of the initial concentration as described above. We thus suggest that the mobilization of a significant portion of intact *S*-MET from the soil microcosms to the water extracts may have contributed at least partially for the high ecotoxicity of the eluates towards the microalgae. Even though *S*-MET levels were not measured in the eluates prepared from the soil samples, using an estimated soil/solution distribution coefficient (*K_d_* value) approximately equal to 2.5 for *S*-MET in a sandy loam soil with 3.1% organic matter [28], it can be anticipated that ∼40% of the *S*-MET present in the soil may be mobilized into the water. Since a soil∶water 1∶10 ratio (w/v) was used in the eluate preparation, this points for predictable *S*-MET concentrations in the water extracts considerably higher (about 50-fold) than the value 8 µg L^−1^ reported as the acute 72 hours median effective concentration for *P. subcapitata* growth [8], or of similar order of magnitude of the MET concentrations reported recently as causing almost complete inhibition of the growth of this microalgae species [6]. According to published toxicological data, phytoplankton species are important targets for damage caused by *S*-MET and other chloroacetanilide herbicides, being more susceptible than aquatic organisms from higher trophic levels [6], [8], [21], [23]. In addition to the specific mode of action of these compounds in the target plants (inhibition of fatty acids synthesis) [23], they also have potential to cause non-specific toxicity over diverse soil and aquatic non-target organisms, including microorganisms, related with the lipophilic nature of their molecules [20], [21], [23]. Since water extracts obtained from the soils contaminated with equivalent quantities of pure *S*-MET were as highly inhibitors of microalgae growth as those prepared from the soils contaminated with Primextra S-Gold, the high ecotoxicity of the latter may be less likely attributable to the presence of ingredients from the commercial formulation other than *S*-MET (and/or the *S*-MET derivatives herein found to accumulate in the soil microcosms, which could also be detrimental for the microalgae). Nevertheless, possible toxic effects of unknown ingredients should not be ruled out as they might have been hidden due to the high toxicity exerted by *S*-MET (and/or derivatives).

The metabolites *S*-MET ESA and *S*-MET OA did build-up in the soil microcosms concomitantly with the moderate dissipation of *S*-MET down to 60% of its initial value. MET can suffer biodegradation in soil [8], [25], [26] and in aquatic systems [29] to form these major metabolites, among others [26], [30]. The main route of chloroacetanilides degradation in soil has been reported to be microbial [26], [29], [30]. For example, ESA and OA derivatives of chloroacetanilides were reported to arise primarily from aerobic dechlorination via GST-mediated reactions and further metabolism of glutathione conjugates by diverse soil bacteria such as pseudomonads and *Enterobacteriacea* [29], [30]. We speculate that under the conditions used in the present work some indigenous active bacteria presumably present in the soil may have contributed for the formation of these two metabolites during the time-course of the biodegradation experiments. Their relative values measured in the soil samples (approximately in a 35∶20 ratio OA∶ESA) are within the range of published values for other soils [26].

ESA and OA degradates of chloroacetanilide herbicides [8] are frequently detected in ground and surface waters worldwide, often at higher concentrations than the parent compounds [29], [31], and are considered of potential concern [31]. Recently, Gadabgui et al. [32] performed a toxicological risk assessment of acetochlor, alachlor and the respective ESA and OA metabolites, and concluded that the toxicity of the degradates for mammals may be lower than that of the parent compounds [32]. To our knowledge scarce information exists about the toxicological properties of ESA and OA metabolites for microorganisms and aquatic organisms. We suggest that the *S*-MET ESA and OA that accumulated in the soils examined herein (and possibly other metabolites that may have formed but were not analysed [26]) might also contribute for the toxicity of the soil water extracts to the microalgae *P. subcapitata*. Importantly, the transformation of *S*-MET into these degradates, whose mobility in soil appear to be greater than that of the parental compound [25], [33], suggests that they may have potential to contaminate water compartments and hence impact water quality [31], [32].

In conclusion, the results herein presented point to the complexity of bioremediating soils contaminated with mixtures of pesticides, in this particular case of ATZ and *S*-MET. Both active substances have been frequently used alone or combined in herbicidal formulations [1], [8], [10], [26]. Due to the potential effects of these substances and their metabolites for non-target aquatic organisms, risk mitigation measures are recommended particularly when the herbicidal formulation is applied in regions with vulnerable soil [31]. To date, as far as we are aware of, effective biodegradation and detoxification of MET/*S*-MET or derivatives ESA and OA in soil have not been well succeeded, apparently because microorganisms do not easily metabolize their aromatic ring [1], [19], [34]. In the present work, there are evidences of the transformation of 40% initial *S*-MET in the contaminated soils, presumably performed by intrinsic microorganisms present in the soil, but this is not associated with an effective decontamination as discussed above. Further work is needed for the development of efficient bioremediation strategies for land contaminated with herbicide mixtures such as the one herein examined. It is necessary, in one hand, to focus on the isolation and optimization of performance of soil bacteria (most probably working in consortia) able to concomitantly biodegrade multiple chemicals to less toxic derivatives; and, in the other hand, to address the effects of multiple pesticides on the performance of specific degrading microorganisms [1], [35]. The present work is a contribution to enhance the knowledge on the latter issue, particularly with respect to the interaction of *S*-MET with the well-known ATZ-degrading bacterium *Pseudomonas* sp. ADP [14]–[18]. Overall results also highlight the importance of monitoring the efficacy of the soil clean-up processes based on ecotoxicity assessments of soil aqueous extracts before and after the implementation of the bioremediation treatment, besides chemical analysis [14]. Ecotoxicological evaluation provide a more realistic glimpse over the ecological risk assessment of soil remediation, being particularly important, in aquatic ecosystems, for the evaluation of the potential impact of the mobilization of extractable and bounded fractions of ATZ, *S*-MET and other pesticides (including their possible metabolites) via water, mainly due to leaching and runoff events.

## Materials and Methods

### Chemicals

Atrazine (ATZ; Pestanal, purity 99.1%), *S*-metolachlor (*S*-MET; Pestanal, purity 98.2%) and 4-morpholinepropanesulfonic acid (MOPS) were purchased from Sigma-Aldrich (Seelze, Germany), trisodium citrate from Merck (Darmstadt, Germany), and [ring-UL-^14^C]Atrazine (purity 99%, specific activity 1.85 GBq mmol^−1^) from American Radiolabeled Chemicals (St. Louis, MO, USA). The formulations Primextra S-Gold (320 g of ATZ L^−1^ and 400 g of *S*-MET L^−1^ as active ingredients; 3 L ha^−1^ as the recommended dose (RD) for weeds in corn plantations) and Atrazerba FL (500 g ATZ L^−1^ as single active ingredient; RD = 2 L ha^−1^) were purchased from Syngenta Portugal (Lisbon, Portugal) and Sapec (Setúbal, Portugal), respectively. The RD of ATZ and *S*-MET (0.96 kg ha^−1^ and 1.2 kg ha^−1^, respectively) were estimated as equivalent to approximately 0.8 and 1.0 µg g^−1^ of soil, respectively, assuming a possible field scenario of herbicide distribution through a 5 cm diameter ×10 cm height soil column and an average soil density of 1.5 g cm^−3^ [15].

### Bacterial strain and culture conditions

A spontaneous rifampicin-resistant (Rif^R^) mutant of *Pseudomonas* sp. ADP which can mineralize atrazine with equal efficiency than the wild-type [36] was used. The cell suspension used as inoculum was prepared from a late-exponential culture (6.5±0.4×10^8^ colony forming units (CFU) ml^−1^, corresponding to OD_640 nm_∼1) grown at 30°C in liquid PADP medium (adapted from [16], [17]) following a procedure reported elsewhere [15]. Briefly, the growth medium was buffered using MOPS (0.1 M; pH 6.2) and supplied with trisodium citrate (10 g L^−1^) as C source [17], and with ATZ (300 mg L^−1^) from Atrazerba FL as sole N source [15]. Cells were harvested by centrifugation (5 min, 9000 *g*), and the cell pellet was washed once and resuspended in sterile saline solution (0.9% w/v NaCl). The concentrated cell suspension obtained was used as inoculum in survival, mineralization and biodegradation experiments, as is described below. The inoculum density, expressed as CFU ml^−1^, was determined by plating culture serial dilutions onto agarized Lennox Broth (LB) medium and further incubation at 30°C during 72 hours.

### Soil parameters

A natural sandy loam soil (pH 6.1; organic matter 3.1%; water content 9.8±0.9%; water holding capacity (WHC) 39.4±1.6%; cation exchange capacity 0.013 cmol g^−1^ soil dry weight; soluble carbon 23.5±5.2 µg g^−1^ soil dry weight) was used [14], [15]. This soil is representative of a corn production field from Central Portugal (Escola Superior Agrária de Coimbra - ESAC, Coimbra, Portugal) with no history of pesticide applications. The soil was sieved (5 mm mesh) and stored in plastic bags at −20°C. Prior to use in the survival, mineralization or biodegradation experiments, soil was defrosted for at least 4 days at 4°C.

### Survival experiments

To examine the effects of *S*-MET on the survival of *Pseudomonas* sp. ADP in ATZ-contaminated soil, experiments were carried out in sterilized EPA vials (40 ml, gastight PTFE/Silicone septa, Sigma–Aldrich) each containing 5 g soil dry weight freshly spiked with ATZ to give an approximate concentration of 24 µg g^−1^ soil dry weight (equivalent to 30×RD of the active substance). Then, three different concentrations of *S*-MET (15, 30 or 60 µg g^−1^) were added to these soils to represent doses equivalent to 15×, 30× and 60× higher than the RD of this active ingredient. Each compound was added from a stock solution in methanol (18 or 20 mg ml^−1^ for ATZ or *S*-MET, respectively), mixed with pure methanol when needed (to guarantee a similar total volume of methanol in all vials) and with sterile deionized water (to obtain an initial soil moisture of 40% soil WHC). Vials non-supplemented with *S*-MET were included as controls. Vials were vigorously stirred in a vortex to promote homogeneous distribution into the soil and were left uncapped in the laminar flow chamber for 1 hour to allow most of the methanol to evaporate. Each vial was then inoculated with an adequate amount of a suspension of bacteria viable cells prepared as described above, in order to have an initial inoculum density of approximately 10^7^ CFU g^−1^ soil dry weight. Vials were stirred again, capped and incubated at 25.1±0.2°C in the dark. The total volume of liquid added to each vial (298 µl; 40% soil WHC) took in account the volumes of herbicide solutions, inoculum and deionized water. In each experiment, 16 replicates of each condition were prepared. To determine *Pseudomonas* sp. ADP Rif^R^ viable cells in soil, at each time interval (up to 9 days) one replicated vial was destructively sampled and processed immediately for the determination of CFU concentration as is described below (microbiological analysis). At least duplicate determinations from two or three independent experiments under identical conditions were carried out.

### Mineralization experiments

ATZ mineralization assays were carried out in sterilized EPA 40-ml vials containing 5 g soil dry weight, as previously described [15] with minor adaptations. Briefly, a mixture of [ring-UL-^14^C]ATZ (stock solution in acetonitrile: 467.7 KBq ml^−1^) plus non-labeled ATZ was incorporated into the soil to give a total activity of 0.65 KBq g^−1^ dry weight of soil and different total concentrations of ATZ in soil. Non-labeled ATZ was supplied from Primextra S-Gold or as the pure substance, depending on the type of experiment to be carried out, as follows: first, to evaluate the effect of increasing doses of Primextra S-Gold in ATZ mineralization by *Pseudomonas* sp. ADP, soil in the vials was freshly spiked with mixtures containing the [ring-UL-^14^C]ATZ stock solution, aqueous suspensions of Primextra S-Gold (to give approximately 5×, 20× and 50×RD corresponding to 15, 60 and 150 L ha^−1^, respectively) and sterile deionized water (to obtain an initial soil moisture of 40% soil WHC). Second, to examine the influence of *S*-MET in ATZ-mineralization bacterial activity, soil in the vials was freshly spiked with mixtures of the [ring-UL-^14^C]ATZ stock solution plus adequate amounts of stock solutions of herbicides in methanol to have total concentrations of 24 or 40 µg of ATZ g^−1^ soil dry weight plus, respectively, 30 or 50 µg g^−1^ of *S*-MET (equivalent to approximately 30× or 50×RD of the active ingredients, respectively) or no added *S*-MET. Pure methanol (to guarantee a similar volume of methanol in all vials) and sterile deionized water (to obtain soil moisture of 40% soil WHC) were also added. Then, soil was vigorously mixed with a vortex apparatus to incorporate the substances, followed by the inoculation with an adequate amount of the bacterial cell suspension prepared as described above (to obtain approximately 10^7^ viable cells g^−1^ soil dry weight) [15]. Non-inoculated controls were included in each set of experiments to account for intrinsic mineralization activity in the soil. In addition, in experiments aiming to examine combination of soil bioaugmentation with biostimulation with citrate on ATZ mineralization, sterile concentrated solutions of trisodium citrate were added to soil to obtain a ratio of C_s_∶N_atz_ equal to 50, as described before [15], [17]. The total volume of liquid added to each vial (298 µl; 40% soil WHC) took in account the volumes added of herbicide solutions, inoculum, a citrate solution and deionized water. After all soil amendments, the vials were stirred once more for homogeneity. Triplicate vials were included for each treatment. A test tube containing 1 ml solution of NaOH 1 M was placed inside each vial to trap the released ^14^CO_2_. All vials were incubated at 25.1±0.2°C in the dark for up to 8 days. At adequate time intervals, the quantity of released ^14^CO_2_ was quantified using a Beckman LS 5000TD scintillation counter, as described before [17]. Ultima Gold (Perkin Elmer, Waltham, USA) was used as the scintillation cocktail in a 1∶4 sample-to-cocktail ratio. At least triplicate determinations from two independent experiments under each condition were carried out.

### Biodegradation experiments at a larger scale

Soil microcosms consisting of glass cylinders (10 cm height×4.5 cm interior diameter) containing 160 g dry weight of soil (∼7 cm×4.5 cm) over a 2 cm height layer of 2-mm-diameter glass beads supported by a fine Teflon mesh were used as described elsewhere [14], [15]. Briefly, the soil was freshly spiked with 5 ml of aqueous suspensions of Primextra S-Gold to obtain approximately 20× or 50×RD (60 or 150 L ha^−1^, respectively). After homogenization with a glass rod to promote incorporation of the herbicides, soil was inoculated with an adequate quantity of the inoculum bacterial suspension (initial density: 2.5–4.1×10^7^ CFU g^−1^ soil dry weight), and either amended or not with trisodium citrate at 1.3 or 3.4 mg g^−1^ soil dry weight in the 20× or 50×RD amended soils, respectively (to give a ratio C_s_∶N_atz_∼50 [15]). Identical experiments with 20× or 50×RD of Atrazerba FL or with pure *S*-MET (25 or 62 µg g^−1^ soil dry weight) instead of Primextra *S*-Gold, were also carried out for comparison purposes. Microcosms non-contaminated with the herbicides or contaminated but not inoculated with the bacterium were also included in each set of experiments as controls. In all experiments, soil moisture was adjusted to 40% soil WHC as described above, taking in account the total volume of liquid (9.5 ml, comprising herbicide suspensions, inoculum, citrate solutions, and deionized water). Amended soils were again mixed, gently packed into the glass cylinders, and incubated at 25.1±0.2°C in the dark for up to 8 days. Soil microcosms were weighted every day to replace the water lost by evaporation with sterile deionized water. Soil samples were collected from the surface, at days 0, 1, 2, 5 and/or 8, and processed immediately for microbiology analysis (1.0±0.3 g dry weight), or stored at −20°C for chemical and ecotoxicological analyses (∼20 g), which were performed as described below. At least two determinations with samples from two independent experiments were carried out.

### Ecotoxicity analysis

Ecotoxicity tests with the model aquatic microalgae *Pseudokirchneriella subcapitata* (strain Nr.WW 15–2521; Carolina Biological Supply Company, Burlington, NC, USA) were carried out on eluates obtained from soil samples collected in the biodegradation experiments (0, 5 and 8 days) as described above. Microalgae culturing procedures were as previously outlined [37]. Soil eluates were prepared following standard methods as described elsewhere [14]. 72-hours *P. subcapitata* growth tests followed standard guidelines [38], [39], on 24-well sterile microplates, at 21 to 23°C and under continuous cool-white fluorescent illumination (100 µE m^−2^ s^−1^). Three 900 µl sub-replicate cultures per replicated eluate, and a standard control with six replicates, were set up and inoculated with 100 µl of algal inoculum, so that the cell density at the start of the tests would be 10^4^ cells ml^−1^. Both pH and conductivity were measured at the start of each test, and measured levels were comparable across treatments and not expected to have deleterious effects on the test organisms [38], [39]. At the end of the 72-hours exposure, algal growth was estimated as the mean specific growth rate (per day). Further details on testing procedures are described elsewhere [37]. For eluates from soil samples taken from non-inoculated microcosms within the same time interval (5 or 8 days), effects of the two doses (20× or 50×RD) of each herbicide application (Primextra S-Gold, Atrazerba FL, or *S*-MET) on microalgae growth were evaluated through one-way analysis of variance followed by Dunnet's test to compare each dose with the respective non-contaminated control soil. One-tailed Student *t*-tests were employed to compare microalgae growth responses between spiked soils that were treated or not with the bioremediation tool, within the same herbicide dose and time interval. The latter tests were also used to assess decontamination efficacy due to bioaugmentation/biostimulation treatments, by comparing microalgae growth in eluates from non-contaminated (controls) and treated contaminated soils, within the same pesticide dose and time interval.

### Microbiological analysis of soil samples

To determine the concentration of CFU of *Pseudomonas* sp. ADP Rif^R^, soil samples from survival and biodegradation experiments were used as a basis for 10-fold dilution series in saline solution (0.9% NaCl w/v) in triplicate. Dilutions were spread plated onto agarized selective LB medium supplemented with rifampicin (50 mg L^−1^) and cycloheximide (100 mg L^−1^). Petri dishes were incubated at 30°C and colonies counted after 96 hours.

### Chemical analysis

Soil samples were thawed at room temperature, dried at 40°C and further processed for analysis of the herbicides and respective metabolites in soil. For analysis of ATZ, DEA and DIA, extracts preparation and analysis by GC-Electron Ionization (EI)-MS (Perkin Elmer-Clarus 500) was carried out as described elsewhere [15]. Recovery range was between 75 and 90%, and the limits of quantification were 25 ng g^−1^ soil dry weight for ATZ, DEA, and DIA. For analysis of *S*-MET and of its OA and ESA derivatives, soil samples were dried at 40°C, extracted three times with a mixture of methanol and water (1∶2) using a Liarre 60 ultrasonic apparatus (15 min) and centrifuged for 15 min at 3000 *g*. Analysis of the combined extracts was performed by GC-EI-MS (Perkin Elmer-Clarus 500) for *S*-MET, and by LC-EI-MS (Agilent 1100 Series) for *S*-MET OA and *S*-MET ESA. All extracts were injected in scan mode to confirm the presence of each analyte and in SIM (single ion monitoring) for quantification purposes. Recovery ranged from 75 and 90% and the limit of quantification was 16 and 6 ng g^−1^ soil dry weight for S-MET and for *S*-MET ESA or S-MET OA, respectively.
